# Vaccine resource tracking systems

**DOI:** 10.1186/1472-6963-14-421

**Published:** 2014-09-22

**Authors:** Katherine Leach-Kemon, Casey M Graves, Elizabeth K Johnson, Rouselle F Lavado, Michael Hanlon, Annie Haakenstad

**Affiliations:** Institute for Health Metrics and Evaluation, University of Washington, 2301 5th Avenue #600, Seattle, WA 98121 USA

**Keywords:** Vaccines, Vaccinations, Immunizations, Resource tracking, Global Vaccine Action Plan

## Abstract

**Background:**

From 1999 to 2010, annual disbursements of development assistance for health for vaccinations increased from $0.5 billion to $2.0 billion (all financial values USD 2010). In its 2012 Global Vaccine Action Plan (GVAP), the World Health Assembly recommended establishing a comprehensive vaccination resource tracking system to better understand the source and recipients of these funds, and ultimately their impact on outcomes. This systematic review aims to respond to the GVAP recommendation in reviewing and assessing the state of the data and literature on vaccination resource tracking.

**Methods:**

We scrutinized all relevant vaccination resource tracking systems identified in the literature and by practitioners in the field. We examined schemes used elsewhere in the health sector and by other sectors. Informant interviews were also conducted to determine what data exists and how it might be utilized. With this information, we completed a qualitative assessment of existing approaches to vaccination resources tracking.

**Results:**

Tracking systems provide information about some vaccine-related activity in the majority of low- and middle-income countries. Data are generally available for the period of 2006–2010. Levels of granularity vary. Interviewees were concerned about the degree of rigor used to validate the data and the lack of verification. Data are often presented in tabular form, which may be unwieldy for non-technical audiences.

**Conclusions:**

The schemes currently in place to track the resources available for vaccinations were fairly advanced relative to other mechanisms in the health sector. Nonetheless, the coverage, validity, and accessibility of vaccination resource tracking data could be ameliorated. Establishing improved feedback loops and verification mechanisms that connect country-level administrators and the international organizations that support reporting efforts would enhance data quality.

**Electronic supplementary material:**

The online version of this article (doi:10.1186/1472-6963-14-421) contains supplementary material, which is available to authorized users.

## 1. Background

In 2010, more than US $2 billion in development assistance for health (DAH) was spent on vaccinations in low- and middle- income countries (all financial values in USD 2010) [1]. This level of spending is the culmination of ten years of rapid growth: in 2000, spending amounted to only approximately US $500 million. This growth corresponds with the creation of the GAVI Alliance (GAVI) as well as the increased support of development assistance partners such as the Bill and Melinda Gates Foundation, the World Health Organization (WHO), and other international organizations.

With this rapid growth in spending in mind, in May 2012 the World Health Assembly recommended the establishment of a comprehensive vaccination resource tracking system, as outlined in the Global Vaccine Action Plan (GVAP) developed by the Decade for Vaccines Collaboration [2]. This landscape analysis responds to that recommendation with an assessment of the strengths and weaknesses of the different tracking systems currently in place. Stakeholders had little information about the validity, accessibility, costs, and coverage of schemes. Our objective was to identify how disparate schemes can be synthesized to achieve the goal of effectively and comprehensively tracking the resources devoted to immunizations. Thus, our aim was to answer the question: How well are vaccination resources being tracked?

## 2. Methods

**Figure 1:**
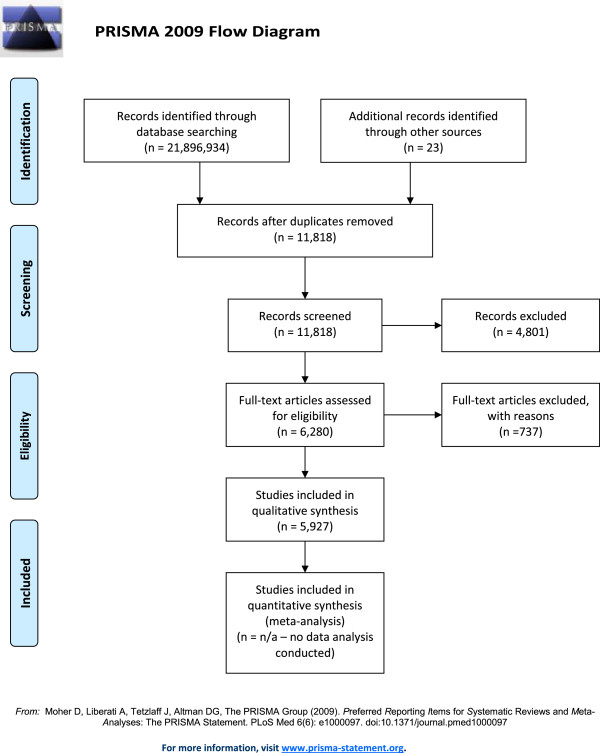
**Review flow diagram.**

We conducted a systematic review of the academic literature, reporting systems, and data sources related to resource tracking in health, with a focus on vaccinations. The following databases were searched: Google (years 2000–2010), Google Scholar (years 2000–2010), and the Global Health Data Exchange (years 2000–2010). We used the following search terms for the Google and Google Scholar searches: “vaccine financing”, “immunization financing”, “immunisation financing”, “vaccine costing”, “immunization costing”, “immunisation costing”, “vaccine funding”, “immunization funding”, and “immunisation funding”. We used the following keywords for a keyword search on the Global Health Data Exchange (http://ghdx.healthmetricsandevaluation.org/keywords): “government health expenditures”, “government health budget”, and “financial assistance for health”.The searches across the three databases yielded 21,896,934 records. An additional 46 sources were identified as potentially applicable as recommended by interviewees. Duplicates across the three databases and 23 of the recommended sources were then removed, leaving 1,349 records. These 1,349 records were screened. A total of 738 were excluded because they pertained exclusively to a time period outside of 2000 to 2010. The remaining 611 records were assessed for eligibility, of which 544 were excluded because no relevant data on vaccine expenditure, supplies or services were available. The remaining 67 data sources were included in the qualitative assessment. Figure 1, the review flow diagram, presents this process.

Tracking mechanisms outside of the health sector were also reviewed to examine whether innovations applied in other areas could be adopted for vaccination resource tracking. In addition to tracking expenditures, our review also considered methods of tracking the physical goods and services required for the delivery of vaccinations. Similar to other development assistance sectors, funds for vaccinations support a complex supply and delivery chain. Many aspects of that chain produce data that can compensate for shortcomings in our understanding of the financing mechanisms.

Data sources and literature were considered relevant if they captured information on the following in low- and middle-income countries: development assistance for health for vaccinations, government spending on vaccinations, non-governmental spending on vaccinations, private sector expenditure on vaccinations, trade in vaccines and vaccine-related supplies (syringes, etc.), purchase of vaccines and vaccine-related supplies, and spending on service delivery for vaccinations. Studies conducted at the global, national, and sub-national level were included. Studies in languages other than English were not considered. However, few mechanisms that operate globally are not provided in English. Studies and data collection were eligible if they took place between 2000 and 2010, as the period relevant to GVAP. When present, however, the full spectrum of years was assessed. The tracking mechanisms operating over this period were assumed to be the most up-to-date and innovative. Data sources and literature were evaluated one-by-one according to the following criteria: costs, coverage, validity and accessibility. The source that best fulfilled the criteria are discussed in the text, while detailed information about all sources evaluated in the qualitative analysis are included in Additional file 1: Data sources by category. Conclusions were drawn from the evaluation of the sources against the criteria.

We conducted informant interviews with experts and practitioners of resource tracking. An expert group of 12 convened by the Decades of Vaccines Collaboration provided recommendations for interviews. Interviewees included representatives from the WHO, the World Bank, the United Nations Children’s Fund (UNICEF), the Sabin Institute, and representatives from a number of ministries of health located in Asia and Latin America. Interviews were unstructured and open-ended, allowing interviewees to freely express their views of the coverage, validity, costs, and accessibility of data. Interviewees were also asked to recommend other vaccine tracking mechanisms of interest. Views were provided by experts fulfilling their roles as staff in international organizations, non-governmental organizations (NGOs), academia, or government. These interviews focused predominately on vaccination resource tracking, but suggestions for tracking efforts to explore throughout health and in other development sectors were also provided.

In addition to in-person and phone interviews, a two-day expert group meeting was convened by the Decades of Vaccines Collaboration in October 2012. Authors presented preliminary findings. Experts provided feedback on the costs, coverage, accessibility, and validity of the data highlighted.

## 3. Results

Different tracking systems focus on different geographic scopes and/or funding agencies, so we segment our review of tracking efforts accordingly. In section 3.1, we examine the three tracking systems which are global in scope. In section 3.2, we examine systems and studies implemented in individual countries. In section 3.3, we examine the tracking of development assistance for vaccinations. In section 3.4, we examine methods to track expenditure based on the service delivery chain. Finally, in section 3.5, we examine how other tracking systems operate and may (or may not) apply to vaccination tracking. The Results section provides an overview of the data and literature most relevant to resource tracking Table 1.

**Table 1:** **Summary of resource tracking assessment**

Tracking scheme	Criteria	Assessment
Joint Reporting Form (JRF)	Coverage	2006 to 2012. Highly aggregated. Spending by NGOs and the private sector not available.
	Validity	Relies on self-reporting. Standardized forms sent to governments on an annual basis. Reports compared by WHO and UNICEF, with some checks on implausible data.
	Accessibility	Spreadsheet. Publicly available. No visualizations.
	Costs	Low but duplication of effort across the cMYPs and APRs in certain years.
Comprehensive Multi-Year Plans (cMYPs)	Coverage	Only one to two years of baseline data available. 72 low- and middle-income countries missing. Highly disaggregated. Spending by NGOs and the private sector not available.
	Validity	Relies on self-reporting. Data provided by governments as part of multiyear planning processes. Little validation.
	Accessibility	Spreadsheet. Publicly available. No visualizations.
	Costs	Low but duplication of effort across the APRs and JRF in certain years.
Annual Progress Reports (APRs)	Coverage	Only for GAVI-eligible countries. Only updated when GAVI requires. Only covers select vaccines. Moderate disaggregation.
	Validity	Relies on self-reporting. Provided by governments as part of applying for further funding from GAVI. Little validation.
	Accessibility	Spreadsheet. Publicly available. No visualizations.
	Costs	Low but duplication of effort across the cMYPs and JRF in certain years.
Sabin Institute’s Sustainable Immunization Financing Program	Coverage	Only covers 18 countries. Government expenditure only. Not an annual, systematized resource tracking process.
	Validity	High given detailed, step-by-step tracking.
	Accessibility	All details not currently available in the public domain.
	Costs	Time and resource intensive
Ad hoc country studies	Coverage	Comprehensive data collection exercise at the country level. Not replicable on a yearly basis on a global scale.
	Validity	More reliable than most sources given the intensive data collection and analytical exercises typically involved.
	Accessibility	In the public domain, generally, but not amassed in one location or systematically reviewed.
	Costs	Time and resource intensive.

### 3.1 Comprehensive systems implemented globally

Across countries, there are three main vaccination tracking mechanisms: the WHO-UNICEF Joint Reporting Form (JRF), the WHO’s comprehensive Multi-Year Plans (cMYPs), and GAVI’s Annual Progress Reports (APRs). All three of these systems track resources in a wide range of low- and middle-income countries and are publicly available through the respective organizations’ websites.

Through the JRF, the WHO, and UNICEF aim to minimize the reporting burden by creating a centralized and standardized national reporting mechanism. The JRF dataset provides information on four vaccination spending components: total expenditure on vaccines, government expenditure on vaccines, total expenditure on routine immunizations, and government expenditure on routine immunizations [3]. The information dates to 2006 and is currently available through 2012. The coverage of the data is extensive: more than 90 low- and middle-income countries have participated in the JRF process. However, our interviews led us to conclude that national and international stakeholders are largely unsatisfied with the process and output. The main issue expressed by stakeholders is that rigorous, consistent verification of the data is generally lacking. When data appear highly implausible, WHO and UNICEF do sometimes reach out to relevant parties to clarify data points. Beyond this ad hoc intervention, many stakeholders reported that little feedback is provided.

The cMYPs, another dataset supported by the WHO, are not designed to be a tracking system per se but rather a tool for countries to plan for investments in vaccination infrastructure and services. The cMYP Immunization Financing Database contains a combination of actual and projected cost and financing data [4]. Government representatives compile the financial information for baseline years. Forecasts for future resource needs are derived from the baseline. The advantage of the cMYPs over any tracking exercise is the granularity of the information. However, a major drawback is that expenditure data are not available beyond the few baseline years. Additionally, as country-based planning documents, the cMYPs are not reviewed intensely by any tracking body. This limits the prospects for making these a part of a global tracking process due to the varying motivations for developing this document. These baseline years are updated sparingly, such that most countries report only one or, at most, two years of expenditure data. Similar to the JRF, stakeholders expressed concerns about the process used to validate the data. The rigor of data collection and the accuracy of information in the database were reported as largely unsatisfactory. Furthermore, the granularity of the data necessitates much more time and resources to compile than that necessary for the JRFs. The high level of detail, however, does make the data more relevant to analyses that disaggregate spending on services versus the vaccines themselves.

Another central source of data is GAVI’s APRs [5]. Countries that receive funds from GAVI are required to submit APRs to the GAVI Secretariat; these reports are used to determine whether funding should continue or if any adjustments to the funding scheme are necessary. The APRs provide information that is similar to, albeit less detailed than, the cMYPs. The APRs provide information on the following categories of expenditure: traditional vaccines, new vaccines, injection supplies, cold chain equipment, personnel, other operational costs, supplemental vaccination activities, and total expenditure. One key shortcoming of the APRs is they do not report on the entirety of spending on vaccinations in each country. They focus on the vaccines and operations supported by GAVI. APRs are available for more countries than the cMYPs, but fewer than the JRF. APRs are required to be submitted to GAVI as supporting documents when applying for further funding, and are thus only updated concurrently with the GAVI funding cycle. Similar to the JRFs and cMYPs, the data have not been rigorously validated according to informants.

Overall, the cMYPs and APRs cover fewer years and countries than the JRF, but are more detailed. Informants suggested that the data validation process for these sources was weak and suppliers of data receive little feedback. Furthermore, timing is another key issue. There is a one-and-a-half-year lag for finalization of the JRF dataset. APRs are updated annually, but also typically suffer from a two-year lag. The cMYPs are refreshed only when a national planning effort takes place, which can occur as far as 5 to 10 years apart.

### 3.2 Country-specific systems

A number of vaccination resource tracking efforts have been conducted at the country level, and these systems generally reflect the organization of a given country’s health system. For example, Waters et al. conducted a study on the coverage and costs of childhood vaccinations in Cameroon [6]. Capobianco and Naido reviewed health sector aid financing in Somalia, including the collection of data on vaccination financing [7]. However, the majority of these exercises appear to be one-off studies that do not provide comparable information on an annual basis. The exception to this is the Sabin Institute’s Sustainable Immunization Financing Program which works in 18 countries across Asia and Africa [8]. With the help of government officials, program staff have followed resources step-by-step through the disbursement process: they track the chain of events from when funding is first allocated, to when ministries of finance and health plan their budgets, to the point at which funds are actually disbursed.

National Health Accounts (NHAs) also collect data on expenditure. Although the 2003 guide for producing NHAs includes a section on vaccination expenditures, our review of more than 900 country-years of NHA data for 120 countries uncovered less than 20 country-years of reported vaccination spending [9]. If NHA data collection processes began to systematically produce this information, these data could be relevant. However, NHAs and similar subaccounts (National AIDS Spending Assessments and Maternal and Child Health Subaccounts) are much more resource intensive than the JRF, cMYPs, and APRs.

### 3.3 Development assistance for vaccinations

More detailed and standardized data are available from organizations that provide development assistance to vaccination programs and the procurement of vaccines. These include bilateral aid organizations, multilateral organizations, private foundations, and others. A number of organizations are dedicated to tracking development assistance, but unfortunately, vaccination spending has not been a major focus of these endeavors. A predominant source of development assistance tracking is the Creditor Reporting System (CRS) database of the Organization for Economic Co-operation and Development (OECD) [10]. In the CRS, vaccination projects are not assigned a specific purpose code and thus cannot be easily identified. Entities like the International Health Partnership, WHO, and ONE also use OECD data to track donor contributions but, again, do not track vaccination financing specifically [11–13]. Another initiative devoted to tracking development assistance is AidData, although no disaggregation of vaccination program funding is currently available [14].

Utilizing the CRS, researchers from the London School of Hygiene and Tropical Medicine (LSHTM) have estimated the immunization funding provided by development assistance partners to 74 developing countries between 2003 and 2010 as part of the Countdown to 2015 Initiative [15]. The estimates relied on hand coding projects, a resource-intensive approach prone to subjectivity. At this stage, further disaggregating these data by country and producing annual estimates would require considerable additional resources. Substantial coverage gaps also exist: Bill and Melinda Gates Foundation support prior to 2009 and GAVI spending prior to 2007 were not included.

Multilateral and bilateral partners, as well as not-for-profit organizations, are also increasingly transparent about their spending. However, large gaps in data still prohibit comprehensive tracking. The most detailed data are provided by the Pan American Health Organization (PAHO), which reports the total value of vaccines procured for each country [16]. UNICEF, in contrast, does not publish information on the value of vaccine procurement for specific countries, but does publish data on total vaccine procurement by antigen and country [17, 18]. GAVI reports timely country disbursement data on its website [19]. Data on vaccine funding channeled by WHO, the WHO-based Global Polio Eradication Initiative, the World Bank, and the Advanced Market Commitment (AMC) are not sufficiently detailed to inform the resource tracking envisioned in the GVAP [20–23].

One of the largest gaps in this category is vaccine financing from NGOs and religious organizations. While some NGOs publish aggregate numbers on total vaccine financing, it is very difficult to determine how much vaccine-specific funding these organizations are spending in specific countries. A substantial amount of resources would have to be committed in order to obtain NGO vaccination expenditure via correspondence.

The DAH database of the Institute for Health Metrics and Evaluation (IHME) has the potential to be one reliable source of data [24]. These data are amassed on a yearly basis from annual reports, government documents, audited financial statements, tax forms, and data sets from public and private donors. The development assistance spending could be drawn out of the current database with the use of keyword search terms and informed review by IHME researchers.

Overall, there are quite a few data sources that include development assistance for vaccinations. However, with the exception of the LSHTM Countdown to 2015 estimates, none of these schemes currently reports information specifically about vaccinations. While this information can be drawn from several of these publicly available databases (CRS, others), resources and time would be required to compile and collate this information. Furthermore, all of these initiatives depend on self-reporting by multilaterals, bilaterals, and, in some cases, NGOs. While these data are generally considered reliable, they are also not cross-validated with country experts. Finally, because development assistance covers only a portion of the vaccine delivery tab, any tracking effort that relied exclusively on these sources would be providing partial information.

### 3.4 Service delivery chain

A number of private and public actors support the supply and service delivery chain associated with vaccinations. Biomedical research firms, wholesalers, distributors, health facilities, and other entities contribute to vaccinations in one way or another. Private actors are also important in low- and middle-income countries. Unfortunately, information about these actors’ behavior is relatively sparse. The most useful data on distribution and procurement is available from UNICEF [17, 18]. It reports detailed data on the number of vaccines and vaccine supplies procured on behalf of GAVI for specific countries. However, the data on total number of doses of vaccines procured by UNICEF is not available at the country level. Commodity data reported in the AMC Annual Reports provides information on the total number of vaccine doses purchased by UNICEF with funds from GAVI and AMC, but these data are not broken down by country [25]. The GAVI procurement commodity data reported by UNICEF could be used to supplement these data. PAHO also procures a large amount of vaccines; this information could potentially be obtained via correspondence but is not generally available [16].

Many low- and middle-income countries do not produce vaccines domestically. Therefore, the United Nations Comtrade and International Trade Center databases can also be used to track vaccine commodities [26, 27]. These databases provide information regarding the number of antigens and other supplies imported.

In terms of information on service delivery, Service Provision Assessment (SPA) surveys could provide data from nationally representative samples of public, private, and not-for-profit health facilities in low- and middle- income countries [28]. SPAs collect information on the provision of vaccination services, the availability and distribution of vaccines by antigen, and cold chain management. SPAs are currently only available for fourteen countries (Bangladesh, Egypt, Ghana, Guatemala, Guyana, Haiti, Kenya, Malawi, Namibia, Rwanda, Senegal, Tanzania, Uganda, and Zambia) over 1997 – 2013 [29].

Overall, any financial tracking exercise could be viably augmented by the good information on procurement and service delivery related to vaccinations. However, again, comprehensiveness is lacking. Poor information about service delivery and procurement in low- and middle-income countries persists, particularly as related to private sector procurement and service delivery [30]. Investment in research and development activities is also a clear gap.

### 3.5 Other tracking mechanisms

To complement our review of vaccination resource tracking, we also conducted an appraisal of tracking mechanisms in other areas of health as well as outside of health. These sources were not included in our systematic review and were only evaluated for comparison purposes. Numerous approaches for tracking disease-specific financing have been pioneered by international agencies and NGOs. Much of this information is produced in a manner similar to the JRF, cMYPs and APRs. However, a few mechanisms incorporate innovations to systems, feedback mechanisms, and presentation of data. The *World Malaria Report*, for example, improves upon data collected directly from donors and standard reporting forms with in-country consultations and household surveys [31]. The *Global Tuberculosis Report* stands out for its introduction of electronic reporting (including built-in, real-time checks) and thorough follow-up with respondents, including country visits and regional workshops [32]. The Every Woman, Every Child Initiative distinguishes its tracking of funds with the use of structured interviews and questionnaires [33].

To ascertain whether lessons could be learned from resource tracking in non-health sectors, we also reviewed a number of those mechanisms. In general, these schemes face challenges similar to those associated with vaccination resource tracking. The non-health resource tracking systems operating at the international level consist mostly of following the disbursement of the publicly available financial resources provided by bilateral and multilateral organizations. The majority of the information is self-reported by governments [34–37]. On the whole, most tracking systems rely on project-based data provided by donors or, in some cases, questionnaires filled out by recipient country officials [38–53]. The lack of oversight and accountability involved in the assembly and provision of much of this data makes for inaccuracy and inconsistencies. There are few systems that employ systematic or data-based checks. They also do not provide a comprehensive picture of non-aid spending on the relevant target.

In terms of tracking the availability of development-assistance related commodities, the sources of data and methods are more readily available than financial data. Maps, reports, and databases provided by the Rome-based agencies and their affiliates are based on household surveys and trade data in addition to questionnaires sent to governments and publicly available data sources [54–56]. The World Food Programme’s Vulnerability Analysis and Map draws predominately from household surveys and employs advanced food security methods to assess current and projected availability of food [54, 57]. The International Food Aid Information System tracks food aid flows based on quantity reporting by donor governments, international organizations, NGOs, recipient countries and World Food Programme (WFP) field offices [58]. WFP reports that these data are cross-checked and continuously updated.

## 4. Discussion

A number of data sources are available to track the funds allocated to vaccinations. These tracking exercises vary according to: coverage, validity, accessibility, and costs. This section discusses the strengths and weaknesses of the data according to these criteria. Overall, coverage of a wide range of countries and years of data are available. However, the validity and accessibility of these data sources are low. Costs vary according to the type of study, but there is clear duplication of efforts across the globally active data collection exercises.

### 4.1 Coverage

In assessing the reporting systems currently operating, we find that the sources cover low- and middle-income countries well. The number of years provided, however, is not very broad and the granularity of expenditure data varies widely across the tracking schemes. The coverage over years is important because tracking schemes, much like surveillance, need to be updated on a yearly basis if they are to be useful. The core vaccination resource tracking mechanisms, the JRF, cMYPs, and APRs, provide data that cover the majority of low- and middle-income countries. These data are of varying levels of granularity. The cMYPs are the source with the finest breakdown of expenditure information, followed by the APRs. The mechanism with the highest level of aggregation, the JRF, still collects information important for monitoring vaccination funding. While the cMYP data are not systematically produced, JRF and APR data are collected annually. The Sabin Institute Sustainable Immunization Financing Initiative as well as ad hoc country studies provide comprehensive, detailed information about the resources available, but are only available for a limited number of countries and few years.

The information provided by the JRF, cMYPs, and APRs allows stakeholders to make decisions at the national level. However, sometimes sub-national differences in expenditure are more important. In contrast, the Sabin Institute’s work duly traces the flow of funds intended for vaccinations across the supply and delivery chain and as they trickle from the national level down to more decentralized units.

Culling feedback on the pertinence of different levels of detail in datasets could strengthen the usefulness of the information. A key finding of this review is that while much is known about the financial resources allocated to vaccinations, not much is known about how these financial resources translate into the supply and service delivery components and, ultimately, seroconversion rates and vaccine effectiveness. The lack of information at the sub-national level, as well as the limited disaggregation in the JRF and APRs, makes these data less useful for managers in the field; these data sources do not provide information that is timely or targeted enough for managers of the supply chain, for example, to assess spending for cost savings or gaps in financial flows. Furthermore, serious gaps exist in information available about private-sector spending, vaccination research and development, procurement, and service delivery.

### 4.2 Validity

The validity of tracking mechanisms, in terms of whether they are a true reflection of reality and thus accurate and dependable, could also be strengthened. The core resource tracking systems depend largely on voluntarily provided data that are not largely validated. This includes the JRF, cMYPs, APRs, and many other development assistance tracking mechanism reviewed. Informants communicated that, due to lack of robust support, data managers have not always been able to conduct data collection processes in a rigorous manner. Data managers are not incentivized to provide highly accurate data nor do they face pressure to improve data if they are of doubtful quality. The lack of checks on most of the reported data leaves room for inconsistencies and inaccuracies. The country studies and Sabin Institute work are likely the most accurate among the tracking endeavors reviewed due to the intensive efforts undertaken to comprehensively collect data at the country level. Vaccination resource tracking could build on the approaches applied in producing the *Global Tuberculosis Report*, where WHO staff invest substantial effort in reviewing data, providing feedback to countries on data quality issues, and promoting tools for in-country validation of results. This includes employing strict online tracking with technological checks on data, person-to-person feedback on implausible data points, and regional workshops or consultations to improve reporting.

### 4.3 Accessibility

For non-technical audiences unaccustomed to handling large amounts of data, the JRF, cMYPs, and APRs do not provide information that can be readily interpreted. Data produced in other tracking schemes has been presented in a way that is easily understandable to all stakeholders. Some tracking mechanisms operating outside of health, such as the Climate Funds Update and Global Humanitarian Assistance, have generated immediately interpretable and targeted visualizations [31, 33]. The vaccination resource tracking mechanisms reviewed do not regularly produce visualization tools, including maps. Furthermore, not all cMYP and JRF data are publicly available; publishing all information collected would improve accessibility.

### 4.4 Costs

It is important that systematic resource tracking endeavors are not burdensome to those who provide vaccinations and do not divert resources away from administering vaccines. The Sabin Institute’s work and the ad hoc country studies are generally much more time and resource intensive. The JRF, cMYPs, and APRs, meanwhile, require fewer resources. The cMYP, however, poses a substantial cost in countries with decentralized health systems or with a substantial number of development assistance partners. One way to reduce costs would be to streamline the data collection occurring across the JFR, APRs, and cMYPs and eliminate duplication.

## 5. Conclusion

This review shows that a number of concerted efforts track the resources allocated to vaccinations. Relative to other areas in health, information about these funds and the services and supplies required for delivery is fairly advanced. However, there is room for improvement across all of the criteria.

The coverage, validity, and accessibility of vaccination resource tracking data could be improved with a number of changes to the tracking systems currently operating. First, streamlining data collection processes and eliminating duplication would reduce the amount of work required of data managers at the country level. This could allow data managers to spend more time and resources focusing on collecting quality data. Once amassed, datasets could be improved with technological checks to flag implausible values as well as feedback and follow-up by the entities tasked with collecting data globally (WHO and UNICEF, for example). Coverage could be improved by enhancing the validity of retrospective datasets. Using a regression model, reported spending could be tied to the delivery of goods and services and coverage rates. With imputation, it may be possible to fill in missing values. Finally, accessibility could be improved by developing tools to visualize the data in different dimensions which are easily interpretable by a wide range of stakeholders.

If tracking mechanisms are to be useful, data must be of high quality and packaged in a relevant, easily understood manner. Their usefulness and application may, in turn, reinforce the need for quality data and thereby encourage rigorous reporting. However, there are clear trade-offs between the resource requirements (both time and money) for highly accurate information and the other criteria. The most reliable data are the most costly to produce.

## Electronic supplementary material

Additional file 1: **Data sources by category.** (DOCX 95 KB)

## References

[1] Institute for Health Metrics and Evaluation (2012). Development Assistance for Health Database (Country and Regional Recipient Level).

[2] World Health Organization (2012). Global Vaccine Act Plan 2011–2020.

[3] World Health Organization (2012). JRF Immunization Financing Indicators Database.

[4] World Health Organization (2012). cMYP Immunization Financing Database. World Health Organization, Immunization Financing Databases.

[5] GAVI Alliance (2012). GAVI Progress Reports.

[6] Waters HR, Dougherty L, Tegang SP, Tran N, Wiysonge CS, Long K, Wolfe ND, Burke DS (2004). Coverage and costs of childhood immunizations in Cameroon. Bull World Health Organ.

[7] Capobianco E, Naidu V (2008). A Review of Health Sector Aid Financing to Somalia. World Bank Working Paper.

[8] **Sabin Vaccine Institute’s Sustainable Immunization Financing Initiative** http://www.sabin.org/updates/pressreleases/sabin-vaccine-institutes-sustainable-immunization-financing-initiative

[9] USAID, World Bank, WHO (2003). Guide to producing national health accounts: with special applications for low-income and middle-income countries.

[10] Organization for Economic Co-operation and Development: **OECD- Creditor Reporting System.** http://stats.oecd.org/Index.aspx?datasetcode=CRS1

[11] World Health Organization (2012). From Whom to Whom? Official Development Assistance for health.

[12] ONE (2012). The 2012 Data Report.

[13] World Health Organization and International Health Partnership (IHP+) (2012). Country Planning Cycle Database.

[14] **AidData database** http://aiddata.org/content/index/data-search

[15] Hsu J, Pitt C, Greco G, Berman P, Mills A (2012). Countdown to 2015: changes in official development assistance to maternal, newborn, and child health in 2009–10, and assessment of progress since 2003. Lancet.

[16] Pan American Health Organization (2009). Financial Report of the Director and Report of the External Auditor, 1 January 2008–31 December 2009.

[17] UNICEF Supply Division (2011). Vaccine Procurement 1996–2011.

[18] UNICEF Supply Division (2012). Supply Annual Report 2011. Supply Annual Report.

[19] GAVI Alliance: **Disbursements by Country.** http://www.gavialliance.org/results/disbursements/

[20] The World Bank: **Trust Funds Database.** https://finances.worldbank.org/Trust-Funds/Trust-Fund-Funding-for-88-IDA-and-former-IDA-Count/x5wq-ricq

[21] World Health Organization (2012). Financial Report and Audited Financial Statements for the Period 1 January 2010–31 December 2011.

[22] *Global Polio Eradication Initiative*. Geneva, Switzerland: Global Polio Eradication Initiative; 2011. http://www.polioeradication.org

[23] GAVI Alliance: **Advanced Market Commitment Documents.** *GAVI Documents* http://www.gavialliance.org/library/gavi-documents/amc/

[24] Institute for Health Metrics and Evaluation (2012). Financing Global Health 2012: The End of the Golden Age?.

[25] GAVI Alliance: **Advanced market commitment documents.** *GAVI Documents* http://www.gavialliance.org/library/gavi-documents/amc/

[26] UN Statistics Division: *United Nations Commodity Trade Statistics Database. UN COMTRADE*. http://comtrade.un.org/db/mr/daCommodities.aspx

[27] International Trade Centre (ITC), Market Analysis and Research: **ITC trade Map.** http://legacy.intracen.org/marketanalysis/TradeMap.aspx

[28] Fort A (2010). Service Provision Assessment.

[29] Measure DHS: *Demographic and Health Surveys*. http://www.measuredhs.com/What-We-Do/survey-search.cfm?pgType=main&SrvyTp=type

[30] Levin A, Kaddar M (2011). Role of the private sector in the provision of immunization services in low- and middle-income countries. Health Policy Plan.

[31] World Health Organization (2012). World Malaria Report 2011.

[32] World Health Organization (2012). Global Tuberculosis Report 2012.

[33] Commission on Information and Accountability (2012). Every Woman, Every Child.

[34] Stasio K, Polycarp C, Ballasteros A, Easton C (2012). Summary of Developed Country “Fast-Start” Climate Finance Pledges.

[35] Climate Funds Update (2012). Climate funds update.

[36] **Budget4change** http://www.budget4change.org/

[37] *Global Humanitarian Assistance*. http://www.globalhumanitarianassistance.org/

[38] United Nations Framework Convention on Climate Change (2014). Finance Portal for Climate Change.

[39] European Community Humanitarian Office (ECH): *Development and Cooperation- EUROPEAID*. http://ec.europa.eu/europeaid/work/funding/beneficiaries

[40] Office for the Coordination of Humanitarian Affairs: *Financial Tracking Service (FTS)*. http://fts.unocha.org/

[41] Development Initiatives, United Nations Economic Commission for Africa, and Africa Partnership Forum (2012). Commit 4 Africa.

[42] *International Aid Transparency Initiative*. 2012. http://www.aidtransparency.net/

[43] World Bank, and Forest Carbon Partnership Facility (FCPF) (2012). FCPF Dashboard.

[44] United Nations Development Programme (UNDP) (2012). UN Multi-Partner Trust Fund Office Gateway.

[45] United Nations Framework Convention on Climate Change (2012). Submissions on Information from Developed Country Parties on the Resources Provided to Fulfil the Commitment Referred to in Decision.

[46] United Nations Educational, Scientific and Cultural Organization (UNESCO): **UNESCO institute for statistics: data centre.** http://stats.uis.unesco.org/unesco/TableViewer/document.aspx?ReportId=143&IF_Language=eng

[47] The World Bank Group: **Edstats Projects Database.** http://ddp-ext.worldbank.org/ext/EdStats/ProjectByProjects/default.html

[48] **DevEx** http://www.devex.com/en/projects

[49] **AidData** http://aiddata.org/content/index/data-search

[50] United States Agency for International Development (USAID): **United States Overseas Locans and Grants (Greenbook).** http://gbk.eads.usaidallnet.gov/

[51] REDD+ Partnership (2011). Voluntary REDD+ Database.

[52] UNESCO, World Education Forum (2012). Dakar Framework for Action, Education for All: Meeting Our Collective Commitments.

[53] The REDD Desk: **REDD Countries Database.** http://www.theredddesk.org/countries

[54] Integrated Food Security Phase Classification (IPC) Global Partners (2012). WFP Vulnerability Analysis and Mapping (VAM).

[55] Food and Agriculture Organization of the United Nations (FAO): **FAOSTAT.** http://faostat.fao.org/?lang=en 6086142

[56] Food and Agriculture Organization of the United Nations (FAO): *International Food Aid Information System (INTERFAIS)*. http://www.wfp.org/fais/

[57] IPC Global Partners (2008). Integrated Food Security Phase Classification Technical Manual, Version 1.1.

[58] World Food Programme: **International Food Aid Information System (INTERFAIS).** http://www.wfp.org/fais/

[CR59] The pre-publication history for this paper can be accessed here:http://www.biomedcentral.com/1472-6963/14/421/prepub

