# Supplementary material for: Vaccine resource tracking systems
Source: BMC Health Serv Res. 2014 Sep 22;14:421. doi: 10.1186/1472-6963-14-421 (PMC4263052; doi:10.1186/1472-6963-14-421)
Supplement: Supplementary file 1 — Additional file 1: Data sources by category. (DOCX 95 KB) [file 12913_2013_3532_MOESM1_ESM.docx]

# Appendix: Data sources by category

## Categorization of data sources

## We have categorized our review of the data into two sets of categories: financial flows and resources flows (covering the physical goods and services required to deliver vaccinations). Financial flows are categorized into sources, channels and agents. Sources are defined as revenue streams of funds. Examples of sources include national treasuries and private individuals. Channels are institutions though which funding is passed or “channeled.” Organizations with the primary purpose of providing development assistance are generally standalone channels. Agents are entities which pool health resources collected from different channels, including actors that pay for health care from their own funds, such as households and firms. Some of the sources cover both sources and channels, or both channels and agents, and those are thus represented in separate categories.

## Resource flows are categorized into: producers, distributors, commodities in country, vaccine and service delivery. Commodities include the vaccines themselves but also the syringes and other supplies required to administer vaccinations. In many lower-income and lower-middle income countries, production of vaccines and vaccination-related supplies would be done abroad, thus why producers and distributors are lumped into separate categories. Commodities would generally be imported and, in many cases, purchases are facilitated by multilateral development partners. Service delivery is distinguished from commodities because the relevant services are not generally traded. Service delivery is further segmented into vaccine delivery and service delivery. Service delivery includes: human resources, program management, training, social mobilization and disease surveillance. Vaccine delivery covers the expenditure on up-front investment and maintenance of cold chain equipment, cold chain overheads, vehicles and transport. These two categories could also be lumped into one classification: service delivery. We also include a special category for data on vaccination coverage, which could be connected to financial and resource flows to determine the ultimate impact of support provided to vaccinations. Finally, consideration of costs is not included in this section; we believe the general assessment found in the body of the text suffices.

## 1) Financial Flows: Sources

Overview:

The annual *PhRMA Industry Profile* provides data on total pharmaceutical sales to countries and regions around the world gathered through the *PhRMA Annual Member Survey*, but data on total vaccine sales is not available [1-2]. The total sales data are only available for a small number of countries in the framework, while other countries are grouped into regions. Compared with the other data sources listed in this section, the G-Finder data are more comprehensive as they report detailed expenditure data tabulated by disease, product, recipient, recipient country, and funder. The data are collected using surveys and by extracting data from the US National Institutes of Health (NIH) grant data. However, the G-Finder data suffer from issues of completeness as the sampling frame has expanded over time and subjects do not always respond to the survey.

Coverage:

No data source is publically available for the entire period of interest (2007 through 2012). The G-Finder database and tax forms are only available through 2010 at the time this document was written, while sales data from the *PhRMA Industry Profile* is only available for years 2009 to 2010*.* Pharmaceutical companies’ published data on charitable giving are available for more recent years. However, accessing historical charitable giving data on pharmaceutical companies’ websites is sometimes problematic. All data in the “Sources” category are updated on an annual basis.

Accessibility:

The G-Finder database is the most accessible data reviewed in this section as the database is easily queried and exported into Excel. The other data sources would be time-consuming to extract as the data would need to be copied and pasted into a spreadsheet to facilitate analysis.

Reliability:

Among those data sources that exclusively capture sources of funding, data quality is low. While data on the value of pharmaceutical vaccine donations exist, the data provided by pharmaceutical companies on their websites is neither disaggregated by recipient country nor consistently disaggregated by vaccine type. Tax forms from pharmaceutical companies’ foundations sometimes report cash grants for vaccine-related activities along with the recipient countries and institutions receiving the grants, but these data are not systematically reported.

Comparability:

The G-Finder Database and *PhRMA Industry Profile* data are collected using standardized methodologies to ensure comparability across years. In contrast, pharmaceutical companies’ published charitable giving data and grants data from pharmaceutical company foundation tax forms is not reported in a standardized manner across companies.

Data gaps:

Data on pharmaceutical companies’ sales of vaccines, vaccine donations, and charitable giving to vaccination programs in GVAP focus countries is not detailed or standardized enough to be useful for a comprehensive analysis of immunization financing. The G-Finder data are useful for tracking R&D funding for vaccines across disease groups, but these data also have limitations due to lack of completeness over time.

| Source | Description | Geographic scope | Years available |
| --- | --- | --- | --- |
| G-Finder database [1] | Provides financial data on research and development expenditures on neglected diseases tabulated by disease, product, recipient, recipient country, and funder. The data are collected via survey and review of US NIH grant data. Completeness of the data is a problem due to survey non-response and the fact that the sampling frame has expanded over time. | Global—Only the country in which the recipient research institution is based is available, not the countries that the vaccines would benefit | 2007-2010 |
| *PhRMA Industry Profiles* [2] | Using data collected through the *PhRMA Annual Member Survey*, the reports provide financial data on total pharmaceutical sales by region and total sales for selected middle- and high-income countries. Data for vaccine sales by country are not available. | Global—some sales data are reported for individual countries while others are grouped by region | Sales data for 2009, 2010 and 2011 available |
| Pharmaceutical companies’ published data on charitable giving | Leading pharmaceutical companies typically post data on their charitable giving on their websites and in annual corporate social responsibility reports. The value of vaccine donations is not always reported separately from other types of pharmaceutical donations. The reported value of product donations may be significantly higher than the value of the product in the recipient country. Furthermore, the quality of these product donations has not been independently assessed. | Giving to individual countries not reported separately, but only as lump sums | 2011, availability of historical data varies by company |
| Pharmaceutical companies’ foundation tax forms available through the Foundation Center [3] | On their tax forms, corporations’ foundations report their charitable donations. Data on pledges, project descriptions, recipient name, and target country and are sometimes available. These tax forms do not capture the value of product donations. As standardized reporting on individual grants is not required by the IRS, data are not available for all pharmaceutical companies’ foundations. For example, the Merck Company Foundation systematically reports pledges to support vaccination programs, but Pfizer and GlaxoSmithKline Foundations do not. | Global—donations to individual countries are not available for all donors | 2000 through 2011 |

## 1-2) Financial Flows: Sources/Channels

Overview:

The OECD Creditor Reporting System (CRS) contains commitment and disbursement data from Development Assistance Committee (DAC) member countries, two non-DAC members (Kuwait and the United Arab Emirates), BMGF, and multiple multilateral funders [4]. In addition to financial data, the OECD-CRS contains detailed project descriptions and information about the channel receiving the money as well as the recipient country. Data reported by Kuwait, the United Arab Emirates, and BMGF are only available for more recent years.

While AidData incorporates data from the OECD-CRS, its prime advantage is that it contains data on assistance from over 20 donors that are not members of the OECD-DAC. The researchers obtain data on non-DAC donors’ foreign assistance from a variety of sources such as project documents, donors’ websites, and directly from donors themselves. They convert the data to a standardized format and manually apply activity codes based on review of project titles and descriptions [5]. AidData researchers have applied activity codes to projects from non-DAC donors to facilitate rapid analysis of projects by focus area.

Foreign assistance data reported to the International Aid Transparency Initiative (IATI) are detailed, project-level data that are reported in a manner similar to that of the OECD-CRS [6]. The level of detail included in project descriptions varies across donors and organizations. Currently, the data reported by UK-based NGOs are largely incomplete as most only report data on DFID-funded projects.

The grants data in the BMGF Online Grants Database and the tax forms is highly disaggregated and detailed [7-8]. However, the Online Grants Database only contains commitments and lacks the disbursement information that is included in the tax forms. Both sets of data lack information on the countries that the grants are targeting, but the Online Grants Database includes information on the region served. BMGF reports detailed data to the OECD-CRS, but the data in the Online Grants Database is timelier and covers a larger number of years.

Coverage:

The data sources that contain data relevant for the time period of the GVAP analysis include the OECD-CRS, AidData, BMGF Online Grants Database, and BMGF tax forms. The OECD-CRS data and BMGF tax forms are available up to 2011 at the time of writing, while the BMGF online database is current through 2013. The time periods covered by AidData and IATI Registry data vary by donor and by organization.

The OECD-CRS database and BMGF tax forms are updated annually. BMGF Online Grants Database is continuously updated, while data in the IATI Registry are updated according to the time schedule determined by each donor or organization. AidData is updated continuously, but these data are not always complete or cited properly. AidData publishes an annual “research release” which corrects for completeness and citation concerns.

Accessibility:

Data from the OECD-CRS and AidData can be quickly exported into a variety of formats suitable for analysis. In IATI, each “activity file” containing grants must be downloaded individually, which is time-consuming. Alternatively, code can be written to download or “webscrape” data files automatically from the IATI Registry.

Data from the BMGF online database cannot be exported in spreadsheet form. Adding this functionality to the database would eliminate the time required to copy and paste the data into a spreadsheet for analytical purposes. Furthermore, the online database lacks disbursement data, but these data are located in BMGF’s tax forms. It is difficult and time-consuming to extract disbursement data from PDFs, so it would be best if BMGF could incorporate its disbursement data into the online database. Also, it would save data users time if BMGF added functionality to its Online Database to permit the data to be downloaded in spreadsheet format.

Reliability:

The data from the OECD-CRS, AidData, and the IATI registry are not systematically validated at the country level. While donors may state that they are devoting a certain amount of money to fund vaccination programs in a specific country, there is a lack of transparent tracking systems in place to monitor how much money actually reaches that country. Furthermore, it is not always possible to discern from project descriptions the types of vaccines donors are funding.

Over time, the reliability of the data found in the CRS has improved. To avoid double-counting, multilaterals do not report earmarked funds received from bilateral donors. From a vaccine resource tracking perspective, it may be best to track multilateral assistance through these organizations’ project databases or financial reports instead of through the CRS in the interest of capturing both core and earmarked funding closer to its ultimate point of disbursement. However, for the GAVI Alliance, we recommend using the data that they report to the OECD-CRS as it includes total expenditures from the organization whereas the financial data published on GAVI’s website only captures a subset of total expenditures. This complete dataset could be potentially obtained from GAVI itself.

Comparability:

The data within OECD-CRS data are comparable since donors report their aid data in a standardized manner using the CRS Directives [9]. The data in the IATI registry are also reported according to the “IATI Standard” [10]. Unlike the OECD-CRS, the data in the AidData database are not reported by donors in a standardized manner, but are compiled by AidData researchers. Therefore, the AidData database is not as comparable as the OECD-CRS data since the data are not reported by donors according to a standardized set of guidelines. The data from the BMGF Online Grants Database and BMGF’s tax forms differ from the BMGF data in the OECD-CRS database due to different reporting systems.

Data gaps:

There are is a plethora of data in the sources/channels category. Currently, the largest gap in this category is data on Chinese foreign assistance. Collaboration with the Peking University Institute for Global Health, who published “China health aid to Africa” in 2011, could be a possible avenue for obtaining data on Chinese funding of immunization programs in developing countries [11].

Unlike the many DAC bilateral organizations that report to the IATI Registry, complete, project-level data on US bilateral disbursements is not yet available past 2011. However, Secretary of State Hillary Clinton’s announcement that the US would join IATI indicates that these data are likely to be available in the near future [12].

Another step towards filling data gaps in this category would be to add disbursement data to the BMGF Online Grants Database. While the database is timely, the utility of the data would be increased through the addition of this important variable.

| Source | Description | Geographic scope | Years available |
| --- | --- | --- | --- |
| OECD-CRS [4] | Contains project-level data from DAC members, Kuwait, United Arab Emirates (UAE), BMGF, and selected multilateral donors. Donors report information such as commitments, disbursements, project descriptions, sector, channel, and recipient country. Data quality varies by donor and has improved over time. Data from Kuwait, UAE, and BMGF are not available for all years. | Global | 1973-2011; not all donors/organizations provide data for entire time period |
| AidData [5] | Contains foreign assistance data from the OECD-CRS, the IATI Registry, project documents, donors’ websites, and project-level data obtained via correspondence. AidData contains data from many donors not captured in the OECD-CRS database. | Global | Varies by donor/organization |
| IATI Registry [6] | Data from 92 bilateral and multilateral donors and UK-based NGOs are represented in this database. The largest bilateral donor, the US, does not yet report its data. | Global | Varies by donor |
| BMGF Online Grants Database [7] | Contains project descriptions, commitments, recipient information, region served, program, and project length. | Global | 1995 onwards |
| BMGF tax forms [8] | Contains project descriptions, disbursements, and recipient information. | Global | 2001 through 2011 |
| London School of Hygiene and Tropical Medicine’s Countdown to 2015 Initiative [9] | Contains estimates of the immunization funding provided by development assistance partners to 74 developing countries. | Global | 2003 through 2010 |
| Institute for Health Metrics and Evaluation’s Development Assistance for Health database [10] | Estimates of development assistance for health by donor and recipient can potentially be pulled from dataset. Not currently publicly available. | Global | 1990 through 2012 |

## 2) Financial Flows: Channels

Overview:

The Advanced Market Commitment Annual Reports report highly aggregated data [15]. While useful for understanding how much money AMC is obtaining from different donors, the expenditure data reported in these documents is only useful for understanding how much funding AMC is disbursing annually to the UN Supply Division to purchase pneumococcal vaccines.

The project databases from the World Bank and the regional development banks report detailed information on commitments and disbursements as well as the recipient countries and implementing agencies [16-20]. The African Development Bank Project Database only reports disbursements for closed projects, however [19]. The World Bank Projects and Trust Funds Databases lack project descriptions, and the Trust Funds Database does not contain data on implementing agencies [16-17].

On its website, the GAVI Alliance makes detailed income and commitment and disbursement data available [19]. The income data are disaggregated by donor and year, while the commitment and disbursement data are tabulated by country, year, and high-level and sub-categories. However, these data only represent a subset of total expenditure, whereas the data that GAVI reports to the OECD-CRS represents total expenditures [4].

Coverage:

AMC Annual Reports cover the all years of the AMC’s existence and are published annually. Data from the World Bank and the regional development banks are constantly updated. GAVI income, commitment, and disbursement data are current as of 2012 and are consistently updated.

Accessibility:

The data from the World Bank can be easily exported into spreadsheet format, but the data from the regional development banks is not exportable. Data from the AMC Annual Report must be extracted from PDFs. GAVI income, commitment, and disbursement data can be downloaded in Excel format.

Reliability:

Data are not systematically validated at the country level; while donors may state that they are devoting a certain amount of money to fund vaccination programs in a specific country, there is no tracking system in place to monitor how much money actually reaches that country.

Comparability:

While they are all gathered through different data collection systems, project databases from the World Bank and the regional development banks are roughly comparable. Each of their systems captures data in a similar manner, with a few important exceptions as mentioned previously. Since the World Bank Trust Fund and Project Databases lack project descriptions, however, it is more difficult to identify projects that fund immunization programs when using these databases.

Data gaps:

Given that the World Bank Project Database does not contain project descriptions, these data should be used in conjunction with the data that the World Bank reports through the IATI Registry as it contains project descriptions.

Since the disbursement data published in the AMC Annual Reports is highly aggregated, researchers could explore the possibility of obtaining more detailed data on the value of purchases of pneumococcal vaccines by country.

While the data reported on the GAVI Alliance’s website is timelier than the data it reports to the CRS, it appears to be only a subset of total expenditure. Researchers could inquire about the possibility of accessing more complete and current data from GAVI that is comparable to what it reports to the OECD-CRS.

| Source | Description | Geographic scope | Years available |
| --- | --- | --- | --- |
| Advanced Market Commitments Annual Reports [15] | Income and expenditure data are reported in the AMC Annual Reports. Income data are reported by donor, while payments to UNICEF Supply Division for pneumococcal vaccine purchases are disaggregated by year and by funding source (GAVI versus AMC). Data on supply commitments are tabulated by pharmaceutical company, number of doses, trial price, supply start date, and the amount of AMC funds that are allocated. Funding data are not disaggregated at the country level. | Global | 2010 onwards |
| World Bank Projects Database [16] | Project database contains project titles, commitments, disbursements, lending instrument, sector and theme codes, project dates, approval and closing date, borrower, and implementing agency. | Global | 1947 onwards |
| World Bank Trust Funds Database [17] | Data capture income received by trust funds disaggregated by donor name and agency, trustee fund name, and fiscal year; a separate database containing trust fund data contains commitment and disbursement amounts; fiscal year; recipient country; agreement date; and program, trustee fund, and grant fund name. | Global | 2007 onwards |
| Asian Development Bank Projects Database [18] | Online database provides project titles and descriptions, recipient country, executing agency, start date, closed date, fund name, and disbursement data. | Asia | 1968 onwards |
| African Development Bank Projects Database [19] | Online database provides project titles and descriptions, recipient country, approval date, start date, completion date, and funding source. Disbursement data are only available for closed projects. | Africa | 1981 onwards |
| Inter-American Development Bank Projects Database [20] | Online database provides project titles and descriptions, recipient country, executing agency, approval date, completion date, fund name, and disbursement data. | Latin America | 1963 onwards |
| GAVI Alliance published data [21] | On their website, GAVI provides income data tabulated by donor and year as well as project-level data. When expenditure data are compared to the data that GAVI reports to the OECD-CRS, however, the data provided on their website appear to be incomplete. | Global | 2000 onwards |

## 2-3) Financial Flows: Channels/Agents

Overview:

UNICEF Vaccine Shipments data and data on vaccine-related procurement from the *UNICEF Supply Division Annual Reports* are highly aggregated [22-23]. The UNICEF Vaccine Shipment data provide data on the overall value of procurement of vaccines by antigen, but these data are not disaggregated by country. The *UNICEF Supply Division Annual Reports* provide the total value of UNICEF’s procurement of pharmaceuticals and other goods for a specific year tabulated by country, but the value of vaccine-specific procurement by country is not provided.

The WHO-based Global Polio Eradication Initiative (GPEI) provides data on the amount of income from different donors for expenditure in years 2012 to 2013 as well as data on total expenditure between years 1988 and 2011 [24]. It is unclear whether the income data they report is commitments or cash received. Detailed information about the amount of expenditure channeled to different countries and spent on oral polio vaccine and service delivery is not available. Disaggregated, country-specific data are only published for budgeted expenditures.

*PAHO Financial Reports* contain data on the amount of funding received from different countries and donors for the purchase of vaccines as well as the amount of money spent on vaccines in a given year [25]. These expenditure data are not disaggregated by type of vaccine.

The 2010-2011 *WHO Financial Report* included a single number for the value of in-kind vaccine donations it received. The annex lists total in-kind donations by donor, but data on vaccination-specific donations are not separated out [26].

In their annual reports and websites, NGOs who carry out vaccine-specific activities often publish data on aggregate expenditure on vaccination-related activities. Comprehensive data on NGOs’ country-specific expenditure is nearly impossible to find in documents published by these organizations.

The USAID Report of Voluntary Agencies (VolAg) and Registry of Private Voluntary Organizations (PVO) contains yearly income and expenditure data reported to USAID by NGOs [27]. The international expenditure data reported by NGOs is neither broken down by country nor by sector. Income data is disaggregated by amount received from the US government and income from private financial contributions, private revenue, and private in-kind donations. Data on the value of total private in-kind revenue received is also reported, but it is not disaggregated by product type.

Coverage:

Most data are published annually, with the exception of the *WHO Financial Reports*, which are published biennially. It is unclear how often GPEI publishes its data.

UNICEF and PAHO data are available up to 2011, while NGO data are only available until year 2010. WHO data are only available for the biennium 2010 to 2011. GPEI expenditure data are available for the period 1988 to 2011, but income data are only available for years 2012-2013. USAID VolAg data are only available through year 2009.

Data from country-level studies are not available for the period 2007 to 2012. The country studies listed in the data sources table were one-time studies.

Accessibility:

All data presented in this section must be extracted from PDF documents. The Institute for Health Metrics and Evaluation (IHME) provides data from the USAID VolAg report in spreadsheet format through its Global Health Data Exchange (GHDx) [26].

Reliability:

The UNICEF, WHO, PAHO and GPEI data are not systematically validated at the country level, although, according to informants, are believed to be more reliable due to the commodities being supplied. Country-level studies are thought to be more reliable due to the resource-intense, detailed nature of the research.

Comparability:

UNICEF Vaccine Shipments data and the *UNICEF Supply Division Annual Reports* are produced using the same accounting system.

Data gaps:

One of the largest gaps in the channel/agent category is data on the amount of immunization funding channeled to developing countries through NGOs and religious organizations. A survey of branches of these organizations operating in low- and middle-income countries could be conducted to quantify immunization-related funding data passing through these channels.

From UNICEF, WHO headquarters, and GPEI, researchers should attempt to obtain data on the value of vaccine-specific expenditure tabulated by country and antigen. Researchers should also ask WHO for data on the value and quantity of in-kind vaccine donations that it channels to different countries. Depending on the timeline for the vaccine financing analysis, the most efficient strategy for obtaining data from WHO may be at the level of its country offices since WHO Headquarters is still in the process of finalizing its country-specific expenditure data for inclusion in the OECD-CRS.

| Source | Description | Geographic scope | Years available |
| --- | --- | --- | --- |
| UNICEF Vaccine Procurement data [22] | Contains total value of vaccines purchased tabulated by type of vaccine. Data are not disaggregated by country. | Global | 1996-2012 |
| *UNICEF Supply Division Annual Reports* [23] | Provide value of total supply procurement tabulated by country. Financing data on vaccine procurement is not available at the country level. | Global | 2000-2012 |
| Global Polio Eradication Initiative Published Documents [24] | The report *Financial Resource Requirements* provides income data on financial resources received by donor for years 2012-2013, and total expenditure from 1988-2011. Expenditure data are not disaggregated by country. The report also includes data on confirmed and tentative funding to specific target countries by donors for years 2012 and 2013. Confirmed and tentative funding is reported by country, year, donor, and expenditure type for years 2012 to 2013 (oral polio vaccine, operational costs, etc.) GPEI also publishes contributions and pledges for years 1984-2014 tabulated by year and by donor. | Afghanistan, Angola, Chad, DRC, India, Nigeria, Pakistan, South Sudan, Sudan | Varied |
| *PAHO Financial Reports* [25] | Provides financial data on income received from countries and specific donors for purchase of vaccines as well as the value of vaccines purchased tabulated by beneficiary country. These data are not broken down by antigen. Data on trust fund disbursements, donors, project descriptions, and recipient countries are also available. | Latin America | At least 1990 to 2012 |
| *WHO Financial Report* [26] | Report cites value of in-kind donations of vaccines and, in annex, lists total in-kind donations by donor (not vaccine-specific). | Global | 2010-2011 |
| NGO published data on program expenditure (5,692 records) | NGOs sometimes provide aggregate numbers on vaccine and vaccine-related program expenditure, but comprehensive country-level expenditure is nearly impossible to find in published documents. | Global | 2007 or earlier-2010 |
| USAID Report of Voluntary Agencies (VolAg) and Registry of Private Voluntary Organizations (PVO) [27] | VolAg reports contain annual financial data reported by NGOs to USAID. Information on in-kind contributions received could be used to estimate how many vaccine donations NGOs are channeling to developing countries. The PVO database could be used to determine which NGOs included in the VolAg report focus on immunization programs. | US-based NGOs and international NGOs receiving funding from USAID | At least 1990 to 2012 |
| Waters et al. Coverage and costs of childhood immunizations in Cameroon. *Bulletin of the World Health Organization* 2004; 82:668-675. [29] | Collected data on immunization expenditures from in-country donors, including bilateral agencies, GAVI, and NGOs. | Cameroon | July 2001-June 2002 |
| Emanuele Capobianco, Veni Naidu. *A Review of Health Sector Aid Financing to Somalia*. Washington, DC: The World Bank, 2008. [30] | Collected data on immunization financing from DAC donors in Somalia via literature review, a survey of donors and implementing institutions, and reviewing donors’ partner contracts. Presents EPI financing by year. | Somalia | 2000-2006 |
| The World Bank; Health, Population, & Nutrition Division. *Immunization Financing in Pakistan.* Washington, DC: The World Bank. 2000. [31] | Presents central and provincial governments’ and external donors’ expenditure on routine EPI by expenditure category (capital goods, vaccines and syringes, salaries, etc.). Expenditure estimates are also available for polio eradication campaigns. Data were collected from the Pakistani government and donors and through discussions with EPI staff. | Pakistan | 1996-1999 |

## 3) Financial Flows: Agents

Overview:

The WHO Immunization Financing Database (cMYP) contains highly disaggregated data on expenditure and budgeted expenditure broken down by detailed expenditure categories such as subcategories of vaccine expenditure, injection supplies, and personnel. In separate tables, expenditure data and budgeted expenditure data are tabulated by secure and probable financing and disaggregated by donor [32]. Based on database documentation, the cMYP database includes data obtained through the *cMYP Costing Tool* and could possibly contain data from GAVI Annual Progress Reports [33-34]. Completeness is an issue with the cMYP as 32 countries included in the framework are missing from the database.

The WHO-UNICEF Joint Reporting Form (JRF) data are gathered through a standardized form that WHO and UNICEF send to country governments [35]. The database contains data on government immunization financing, but it is not as detailed as the WHO Immunization Financing Database (cMYP) since it does not break down expenditure into finer categories beyond vaccines and routine immunization. The JRF database’s reporting of total expenditure on vaccines and routine immunization from all sources may be underreported since NGOs and private sector expenditure on immunization is not widely available.

The GAVI Annual Progress Reports (APRs) report detailed expenditure data by category and sources of funding, but the categories reported are not as detailed as those included in the WHO cMYP data [36]. Unlike the cMYP data, which reports expenditure categories and sources of funding separately, the GAVI APRs report both in the same table.

The UN Comtrade database contains import and export trade value data that are disaggregated by year, country, reporter (exporting country), partner (importing country), and product [37]. Vaccine trade data are only available at the aggregate level, grouped in broad categories such as vaccines for human use and syringes. Unlike the Comtrade database, the ITC database contains values of exported and imported commodities such as vaccines tabulated by year and by the country importing or exporting the goods [38]. While the ITC database is based on the UN Comtrade database, it is more complete than Comtrade as it includes trade data for countries that are missing from the Comtrade database.

Coverage:

Expenditure data from the WHO cMYP database only appear to be available for one year, while budgeted expenditure are available for multiple years. The database covers the period 2004 to 2016, but completeness varies by country.

The government immunization expenditure data from the WHO-UNICEF JRF are available for years 2006 to 2011, while data on immunization expenditure from all sources is available for years 2010 to 2011. The UN Comtrade and ITC databases are available through 2011, while the GAVI APRs are available through 2010.

First launched in 2005, the WHO cMYP database has been updated annually since 2009 [33]. The WHO-UNICEF Joint Reporting Form (JRF), GAVI Annual Progress Reports, UN Comtrade, and ITC databases are updated annually. The country analyses mentioned in the table were one-time studies.

Accessibility:

The WHO-UNICEF JRF and the UN Comtrade and ITC Databases are exportable in spreadsheet format. Data from the WHO cMYP database is also available in spreadsheet format, but files for each country must be downloaded individually. Data from the GAVI Annual Progress Reports must be extracted from PDFs.

Reliability:

The WHO cMYP, WHO-UNICEF JRF, GAVI Annual Progress Reports, and the UN Comtrade and ITC Databases can be used to validate how much donor funding is actually reaching a country’s treasury versus how much donors report channeling to a country. Data reported through most of these systems are not validated, however, and thus could contain errors. The WHO-UNICEF JRF data are compared by WHO and UNICEF headquarters when each agency receives the data from countries, but it is unclear how many potential errors this process could identify.

Comparability:

The country studies by Kaddar et al. and Levin et al. are comparable as they were collected using guidelines published by the Partnerships for Healthcare Reform [39]. The other country-level studies explored below use different data collection methodologies, and are thus not as comparable.

The data in the cMYP include some data from the GAVI ARPs, so some indicators are duplicated across these two sources. Data from the WHO-UNICEF JRF are collected using a different tool and likely not comparable to those data collected through the cMYP database.

The UN Comtrade and ITC databases are comparable in that the ITC database derives much of its data from the UN Comtrade databases, but the ITC data incorporates additional trade data for countries missing from the Comtrade database.

Data gaps:

The most detailed data in the agents’ category are available from the WHO cMYP database. Since a large number of countries are missing from the WHO cMYP and the data are only available for a single year, additional resources could be invested in collecting expenditure data using this tool from more countries and for a larger number of years.

| Source | Description | Geographic scope | Years available |
| --- | --- | --- | --- |
| Waters et al. Coverage and costs of childhood immunizations in Cameroon. *Bulletin of the World Health Organization* 2004;82:668-675. [29] | Collected data on budgeted expenditures on immunization from the Ministry of Health. | Cameroon | July 2001-June 2002 |
| WHO Immunization Financing Database (cMYP) [32] | Contains data reported by countries on actual (“baseline”) and projected expenditures tabulated by type of vaccine-related product as well as funding gaps for these types of expenditure. Countries also report secure and probable financing tabulated by funder (government and donors). Actual expenditure data only appear to be available for one year. | Many low- and middle income countries | Varies by country |
| WHO-UNICEF Joint Reporting Form [35] | Data are available on total expenditure on vaccines, government expenditure on vaccines, total expenditure on routine immunizations and government expenditure on routine immunizations. These data are tabulated by year. | All countries in framework are covered | Government expenditure data: 2006-2011; total expenditure data from all sources: 2010-2011, but completeness varies by country |
| GAVI Annual Progress Reports [36] | Immunization funding data are tabulated by category (traditional vaccines, new vaccines, personnel, etc.) and by source of funding (country, GAVI, UNICEF, etc.). Budgeted expenditure is also provided. | All GAVI-eligible countries | 2001 or later-2011 |
| UN Comtrade Database [37] | Provides value of vaccines imported to countries worldwide on a yearly basis. | Global | 1996-2011 |
| ITC Database [38] | Provides value of vaccines imported to countries worldwide on a yearly basis. | Global | 2001-2012 |
| R. Biellik et al. Health systems and immunization financing for human papillomavirus vaccine  introduction in low-resource settings / *Vaccine* 27 (2009) 6203–6209. [40] | Collected data on government expenditure on immunization programs and the sources (external versus internal) that financed this spending in India, Peru, Uganda, and Vietnam. Data were collected from published literature, stakeholder interviews, and secondary data. | India, Peru, Uganda, and Vietnam | Not reported |
| ARIVA reports [41] | Includes government expenditure on vaccines by financing source. | Burkina Faso, Benin, Cape Verde, Cote d’Ivoire, The Gambia, Guinea, Guinea Bissau, Mali, Mauritania, Niger, Senegal, Chad, Togo | Mid-1990s-2001 |
| Kaddar, Miloud, Vito L. Tanzi and Leanne Dougherty. May 2000. *Case Study on the Costs and Financing of*  *Immunization Services in Côte d’Ivoire.* Special Initiatives Report 24. Bethesda, MD: Partnerships for Health Reform  Project, Abt Associates Inc. [42] | Authors collected immunization cost and financing data via government publications and interviews with staff of the Ministry of Health, Ministry of Financing, private sector, and donors. They calculated the full cost of the National Immunization Program and recurrent costs. | Côte d’Ivoire | 1998 |
| Kaddar, Miloud, Sangeeta Mookherji, Denise DeRoeck and Denise Antona. September 1999. *Case Study on the Costs and*  *Financing of Immunization Services in Morocco.* Special Initiatives Report No. 18. Bethesda, MD: Partnerships for Health  Reform Project, Abt Associates Inc. [43] | Authors collected immunization cost and financing data via government publications and interviews with staff of the Ministry of Health, Ministry of Financing, private sector, and donors. They calculated the full cost of the National Immunization Program and recurrent costs. | Morocco | 1997-1998 |
| Levin, Ann, Sushil Howlader, Sujata Ram, Syed Mizan Siddiqui, Izaz Razul and Subrata Routh. 1999. *Case Study on the Costs*  *and Financing of Immunization Services in Bangladesh.* Special Initiatives Report No. 21. Bethesda, MD: Partnerships for Health  Reform Project, Abt Associates Inc. [44] | Authors collected immunization cost and financing data via government publications and interviews with staff of the Ministry of Health and Family Welfare, private sector, NGOs, and donors. They gathered data on internal and external financing of the EPI program from EPI headquarters, donor databases, and projects that collaborated with NGOs. Data on local government contributions to the national immunization program were obtained via a municipality survey. Furthermore, researchers carried out a survey of private health facilities in the capital to understand the role of the private sector in provision of vaccination services. | Bangladesh | 1997-1998 |
| Asian Vaccination Initiative. *Sri Lanka*  *National*  *Immunization*  *Program* *Financing Assessment*. Manila: Asian Development Bank, 2001. [45] | Data were obtained from published documents and interviews with staff of government and donor organizations at the central and provincial level. Immunization financing and costing data came from data furnished by the central Ministry of Health (Epidemiological Unit and Family Health Bureau), management reports such as the Country Report on the EPI program, work a previous consultant, and UNICEF’s country and global logistics reports. | Sri Lanka | 1999 |
| Chee, Grace. *Cambodia*  *Immunization*  *Assessment: Report of*  *Financial Findings*. Bethesda: Abt Associates, 2000. [46] | Presents expenditure in 1999 by expenditure category (vaccines, operation costs, salaries, etc.) and source (central government, UNICEF, WHO, etc). Financing data were collected using the draft CVP/Abt immunization financing tool. | Cambodia | 1999 |
| Levin, Ann, Sarah England, Joanne Jorissen, Bertha Garshong, and James Teprey. September 2001. *Case Study on the Costs and Financing of*  *Immunization Services in Ghana.* Bethesda, MD: The Partners for Health Reform*plus* Project, Abt Associates Inc.[47] | Researchers developed questionnaires to gather data on immunization costs and funding. They administered these questionnaires at the regional, district, and subdistrict levels. Government documents were also used as a data source. | Ghana | 2000 |
| The World Bank; Health, Population, & Nutrition Division. *Immunization Financing in Pakistan.* Washington, DC: The World Bank. 2000. [48] | Presents central and provincial governments’ and external donors’ expenditure on routine EPI programs by expenditure category (capital goods, vaccines and syringes, salaries, etc). Expenditure estimates are also available for polio eradication campaigns. Data were collected from the Pakistani government and donors and through discussions with EPI staff. | Pakistan | 1996-1999 |
| Sabin Institute’s Sustainable Immunization Financing Initiative [49] | Funds for vaccinations tracked from national to local level. | Bhutan, Cambodia, Cameroon, Congo, Democratic Republic of Congo, Ethiopia, Kenya, Liberia, Madagascar, Mali, Mongolia, Nepal, Nigeria, Senegal, Sierra Leone, Sri Lanka, Uganda, and Vietnam. | N/A |

## 4) Resource Flows: Producers

Overview:

If available, data reported by pharmaceutical companies on the number of vaccine doses donated is typically reported as a single number for a specific antigen. Data on organizations channeling the donations and recipient country are generally not available. Overall, data on vaccine donations published by pharmaceutical companies are too aggregated to be useful.

Coverage & Reliability:

Data are usually reported annually, but pharmaceutical companies sometimes remove historical charitable donation data from their websites.

Accessibility:

Typically, data must be extracted from PDFs.

Data gaps:

Detailed data on the number of doses of vaccines donated and sold to developing countries by pharmaceutical companies is not available. These data could potentially be obtained through a survey of manufacturers.

| Source | Description | Geographic scope | Years available |
| --- | --- | --- | --- |
| Pharmaceutical companies’ published data on charitable giving | Units of corporate vaccine donations are typically reported at the aggregate level. | Global | Varies by company |

## 5) Resource Flows: Distribution

Overview:

The most useful data for tracking resource flows at the point of distribution is the UNICEF Vaccine Shipments data. UNICEF publishes detailed information on vaccine and vaccine supply shipments procured on behalf of GAVI. Tables present the number of doses of different vaccines and units of vaccination supplies shipped to different countries tabulated by product group, such as pentavalent, pneumococcal, and yellow fever. Color coding indicates GAVI-financed and co-financed products, as well as confirmed and unconfirmed quantities.

Unlike the GAVI vaccine supply shipment data, UNICEF’s published data on the total number of vaccine doses procured by antigen is less useful since the data are not tabulated by country.

Compared to the data on UNICEF’s Vaccine Shipments procured on behalf of GAVI, the data from AMC Annual Reports contains much less detail. The data from this source show how many doses of pneumococcal vaccine were financed with AMC funds versus GAVI funds. The reports also contain data on commitments of pneumococcal vaccine doses tabulated by start date, trial price, and pharmaceutical company.

Coverage:

Both UNICEF Vaccine Shipments data and the AMC Annual Reports are updated annually. The AMC Annual Reports are available through the year it began disbursing funds (2010) through 2012, while the UNICEF data are only available through 2012.

Accessibility:

Data from the AMC Annual Reports and UNICEF Vaccine Shipments data must be extracted from PDFs.

Reliability:

Data are not systematically validated at the country level. However, due to the nature of commodities tracking, these data are seen as fairly reliable, according to informants.

Comparability:

Data are reported through different organizations’ accounting systems and at different levels of the distribution chain.

Data gaps and recommendations for addressing them:

Data on the number of doses of vaccines procured through the PAHO Revolving Fund tabulated by country and vaccine type is not publicly available, and the UNICEF data on number of doses of vaccines procured by antigen is not available at the country level. While the detailed data that UNICEF publishes on vaccine-related procurement for GAVI is useful for tracking immunization funding to different countries, researchers should attempt to obtain from UNICEF more detailed data that captures total procurement by country. Researchers should also communicate with PAHO to obtain data on the number of vaccine doses that it procures tabulated by antigen, year, and country.

| Source | Description | Geographic scope | Years available |
| --- | --- | --- | --- |
| Advanced Market Commitments Annual Reports [15] | Reports contain data on total number of doses of pneumococcal vaccine contracted and purchased via UNICEF Supply Division by year. These data show the number of pneumococcal vaccine doses purchased by UNICEF with funds from GAVI and AMC. Data on the number of doses channeled to specific countries is not reported. Data on supply commitments are tabulated by pharmaceutical company, number of doses, trial price, supply start date, and the amount of AMC funds that are allocated. Pneumococcal vaccine contracted doses are also reported tabulated by year. | Global | 2010-present |
| UNICEF Vaccine Procurement Data [22] | Detailed data on vaccine and vaccine supply commodity shipments for GAVI are tabulated by country. UNICEF also provides data on total number of doses of vaccines procured annually by type, but these data are not tabulated by country. | Global | GAVI: 2002-present; UNICEF total: 1996-2012 |

## 6) Resource Flows: Commodities in country

Overview:

The datasets explored in this category are not useful for measuring the total number of vaccine doses and supplies received by different countries. The UN Comtrade database reports the weight in kilograms of vaccines for human use imported and exported to different countries, but data on the number of doses is not available. Also, commodity data on imports and exports by type of vaccine are not available.

GAVI Annual Progress Reports (APRs) provide information on the number of doses of new and underused vaccines and supplies that countries have received. While these data can be used to track bottlenecks in the vaccine delivery system, data on total number of doses of GAVI-funded vaccines received are not captured in the APRs.

Coverage:

GAVI APRs are available through year 2010, while the UN Comtrade data is available through 2011.

Accessibility:

UN Comtrade data can be easily exported to a spreadsheet, while data from the GAVI APRs must be copied and pasted from PDFs.

Reliability:

Data quality audits are performed infrequently to verify the quality of data reported in the GAVI Annual Progress Reports.

Data gaps:

Review of possible sources in the category of “Commodities in country” has revealed that data on the number of doses of specific types of vaccines reaching the country are lacking. One way to address this would be by obtaining data from the WHO-UNICEF Joint Reporting Form, which collects data on the total number of doses of vaccines procured at the national level tabulated by antigen and procuring agency. Data on the number of vaccine doses financed by the government are also collected in a similar format. These data are not currently published by the WHO in the JRF database available online [33,47].

| Source | Description | Geographic scope | Years available |
| --- | --- | --- | --- |
| GAVI Annual Progress reports [36] | On their website, GAVI provides annual progress reports. In these reports, countries provide data on the number of doses of new and underused vaccines and supplies purchased with GAVI funds that they received. Data are tabulated by vaccine type, amount of doses originally approved by GAVI for the year, total doses received, and total doses postponed for the following year. | Global | 2001 or later -2011 |
| UN Comtrade Database [37] | Provides units (weight in kilograms) of vaccines imported to countries worldwide on a yearly basis. | Global | 1996-2011 |

## 7-8) Resource Flows: Vaccine and service delivery

Overview:

Service Provision Assessment (SPA) surveys collect data from nationally representative samples of public, private, and not-for-profit health facilities in low- and middle-income countries.^[48]^ Data collected through SPAs include: provision of vaccination services for children and pregnant women, availability and distribution of vaccines by antigen, storage of vaccines and cold chain management, availability of vaccine supplies, and questions about health facilities’ protocols for ordering vaccines.

Coverage:

SPA surveys are available for fourteen countries over 1997 - 2013: Bangladesh, Egypt, Ghana, Guatemala, Guyana, Haiti, Kenya, Malawi, Namibia, Rwanda, Senegal, Tanzania, Uganda, and Zambia. None of the country studies listed below analyzed data for the time period of interest.

Accessibility:

SPA surveys are available in multiple electronic formats such as SPSS and STATA.

Reliability:

These primary data collection efforts are generally viewed as rigorous, although cross validation may be required to be viewed as fully reliable.

Data gaps:

While SPA surveys are useful for assessing vaccine and service delivery, few of these surveys have been done in recent years. If funding is available, data gaps could be addressed by conducting health facility surveys using the SPA methodology in a larger number of countries.

| Source | Description | Geographic scope | Years available |
| --- | --- | --- | --- |
| Waters et al. Coverage and costs of childhood immunizations in Cameroon. *Bulletin of the World Health Organization* 2004;82:668-675. [29] | Collected data on immunizations administered and stock outs of different types of vaccines from health clinics in three provinces. | Cameroon | July 2001-June 2002 |
| Kaddar, Miloud, Vito L. Tanzi and Leanne Dougherty. May 2000. *Case Study on the Costs and Financing of*  *Immunization Services in Côte d’Ivoire.* Special Initiatives Report 24. Bethesda, MD: Partnerships for Health Reform  Project, Abt Associates Inc. [42] | Authors collected immunization cost and financing data via government publications and interviews with staff of the Ministry of Health, Ministry of Financing, private sector, and donors. They calculated the full cost of the National Immunization Program and recurrent costs. | Côte d’Ivoire | 1998 |
| Kaddar, Miloud, Sangeeta Mookherji, Denise DeRoeck and Denise Antona. September 1999. *Case Study on the Costs and*  *Financing of Immunization Services in Morocco.* Special Initiatives Report No. 18. Bethesda, MD: Partnerships for Health  Reform Project, Abt Associates Inc. [43] | Authors collected immunization cost and financing data via government publications and interviews with staff of the Ministry of Health, Ministry of Financing, private sector, and donors. They calculated the full cost of the National Immunization Program and recurrent costs. | Morocco | 1997-1998 |
| Asian Vaccination Initiative. *Sri Lanka*  *National*  *Immunization*  *Program* *Financing Assessment*. Manila: Asian Development Bank, 2001. [45] | Data were obtained from published documents and interviews with staff of government and donor organizations at the central and provincial level. Immunization financing and costing data came from data furnished by the central Ministry of Health (Epidemiological Unit and Family Health Bureau), management reports such as the Country Report on the EPI program, work a previous consultant, and UNICEF’s country and global logistics reports. | Sri Lanka | Projected EPI costs cover years 2001-2005 |
| Chee, Grace. *Cambodia*  *Immunization*  *Assessment: Report of*  *Financial Findings*. Bethesda: Abt Associates, 2000. [46] | Presents expenditure in 1999 by expenditure category (vaccines, operation costs, salaries, etc.) and source (central government, UNICEF, WHO, etc). Financing data were collected using the draft CVP/Abt immunization financing tool. | Cambodia | 1999 |
| Levin, Ann, Sarah England, Joanne Jorissen, Bertha Garshong, and James Teprey. September 2001. *Case Study on the Costs and Financing of*  *Immunization Services in Ghana.* Bethesda, MD: The Partners for Health Reform*plus* Project, Abt Associates Inc. [47] | Researchers developed questionnaires to gather data on immunization costs and funding. They administered these questionnaires at the regional, district, and subdistrict levels. Government documents were also used as a data source. | Ghana | 2000 |
| The World Bank; Health, Population, & Nutrition Division. *Immunization Financing in Pakistan.* Washington, DC: The World Bank. 2000. [48] | Presents central and provincial governments’ and external donors’ expenditure on routine EPI by expenditure category (capital goods, vaccines and syringes, salaries, etc). Expenditure estimates are also available for polio eradication campaigns. Data were collected from the Pakistani government and donors and through discussions with EPI staff. | Pakistan | 1996-1999 |
| Health facility surveys (such as Service Provision Assessment surveys) [51-52] | Provide information on vaccine services offered at health facilities. | Select developing countries primarily in Africa | Selected years from late 1990s onwards |
| Levin and Kaddar. Role of the private sector in the provision  of immunization services in low- and  middle-income countries. *Health Policy and Planning* 2011; 26:i4–i12  doi:10.1093/heapol/czr037. [53] | Literature review of country-based studies of private sector’s role in vaccination service provision in low- and middle-income countries. | 22 low- and middle-income countries primarily in Asia (Bangladesh, Cambodia, India, Pakistan, Sri Lanka, Ghana, Ethiopia, Kenya, Mauritania, Morocco, Zimbabwe, Honduras, Nicaragua, and El Salvador | Select years during the period spanning from late 1990s to 2005 |
| Ebong and Levy. Impact of the introduction of new vaccines and  vaccine wastage rate on the cost-effectiveness of  routine EPI: lessons from a descriptive study in a  Cameroonian health district. *Cost Effectiveness and Resource Allocation* 2011, 9:9  http://www.resource  allocation.com/content/9/1/9. [54] | Authors gathered data on service delivery costs at health clinics in a district in Cameroon. Furthermore, they collected data on vaccine use and calculated vaccine wastage rates by antigen and delivery strategy. | Cameroon | 2009 |
| Lydon, Patrick. *The Lao PDR Measles Campaigns 2000-2001*. World Health Organization. [55] | Immunization costing data were obtained from financial records (invoices and record keeping) as well as interviews and observations at the provincial health office and a district health office in Borikhamxay Province. Data were also collected from interviews with key informants from the Centre for Mother and Child Health at the Ministry of Health and WHO, UNICEF, and JICA. | Laos | 2001 |

## 9) Vaccination Coverage

Overview:

Data from Demographic and Health Surveys (DHS) and Multiple Indicator Cluster (MICS) surveys are nationally representative and are typically published in the form of microdata [56-57]. WHO and UNICEF Reported Estimates of Immunization Coverage Time Series and WHO and UNICEF Estimates of Immunization Coverage Time Series contain coverage estimates tabulated by antigen, year, and country [58-59]. Annual health statistical yearbooks published by ministries of health typically contain data on national vaccination coverage tabulated by antigen and sometimes the number of doses delivered for each antigen.

Coverage:

The DHS and MICS are carried out about every five years in low- and middle-income countries, while WHO and UNICEF immunization coverage data (both country-reported and WHO/UNICEF estimated) are updated annually. Annual health statistical yearbooks are typically published annually by ministries of health with a one to three year delay.

Accessibility:

DHS and MICS surveys are available in electronic formats such as SPSS and STATA. WHO and UNICEF Immunization Coverage Time Series are published in Excel format [61].

Reliability:

Previous research by Lim and colleagues indicated that immunization coverage data derived from household health surveys is higher quality than coverage data from administrative sources and WHO/UNICEF immunization coverage estimates [60].

Comparability:

According to UNICEF staff, the differing methodology used in DHS and MICS surveys makes it so that their comparability is limited.

Data gaps:

Increasing the frequency of household health surveys that collect data on immunization coverage and other topics to occur every two to three years should be considered as a means of obtaining timelier data for monitoring purposes [60].

| Source | Description | Geographic scope | Years available |
| --- | --- | --- | --- |
| ARIVA reports [41] | Includes vaccination coverage estimates from Ministry of Health | Burkina Faso, Benin, Cape Verde, Cote d’Ivoire, The Gambia, Guinea, Guinea Bissau, Mali, Mauritania, Niger, Senegal, Chad, Togo | Mid-1990s-2001 |
| Household surveys (DHS, MICS, etc.) [56-57] | Provide data on childhood vaccination coverage obtained from mothers’ reports and information from vaccination cards. | Low- and middle-income countries worldwide | Select years over the period 1986-2011 |
| WHO and UNICEF Reported Estimates of Immunization Coverage Time Series [58] | Database contains official estimates of vaccine coverage reported by countries tabulated by antigen, year, and country. | Global | Varies by vaccine type ( depends on year vaccine was introduced) |
| WHO and UNICEF Estimates of Immunization Coverage Time Series [59] | Database contains WHO and UNICEF’s best estimates of immunization coverage based on available data reported by countries and from published and grey literature, evaluation of possible biases, and input from local experts. Data are tabulated by antigen, year, and country. | Global | Varies by vaccine type ( depends on year vaccine was introduced) |
| Annual health statistical yearbooks | Annual publications from ministries of health typically report vaccination coverage rates and sometimes the number of doses delivered tabulated by antigen. | Global | Varies by country |
| Lim et al. Tracking progress towards universal childhood immunisation and the impact of global initiatives: a systematic analysis of three-dose diphtheria, tetanus, and pertussis immunisation coverage. *Lancet*. [Volume 372, Issue 9655](http://www.sciencedirect.com/science/journal/01406736/372/9655), 13–19 December 2008, Pages 2031–2046 [60] | Provides estimates of three-dose diphtheria, tetanus, and pertussis (DTP3) coverage as well as confidence intervals. Estimates are based on data from household surveys. | Global | 1986-2006 |

## 7-8-9) Vaccine and service delivery and vaccination coverage

Overview:

GPEI Polio Campaign Monitoring Reports contain information about the date of the campaign for which the results are being monitored; the number of children, regions, and district targeted; and the scope of the independent monitoring (number of districts monitored, number of children under 5 monitored, number of independent monitors involved, etc) [62]. The results of campaign monitoring are presented as the number of children under-5 with a finger marking indicating vaccination and the number of children without finger markings. Results for both house-to-house monitoring and outside house monitoring are reported. Additional indicators are also reported, such as reasons that children were missed and parents’ awareness of the polio vaccination campaign.

The WHO Measles Supplementary Immunization Activities (SIAs) database contains the following types of information pertaining to SIAs: year, country, type of intervention (measles, MMR, or MR), follow-up or catch-up activity, age group targeted, geographic scope, number of persons targeted and number of persons reached [63]. The methodology used to calculate number of persons reached is not described on the WHO website.

Coverage:

The frequency with which GPEI Polio Campaign Monitoring Reports are collected is unclear. The dates covered by these reports vary by country and span the period 2009 to 2011. The WHO Measles SIAs database is updated annually and contains data through 2011. None of the country studies listed below analyzed data for the time period of interest.

Accessibility:

GPEI Polio Campaign Monitoring Reports must be extracted from PDFs. The WHO Measles SIAs database is published in Excel format [63].

Reliability:

As noted in the previous section: research by Lim and colleagues indicated that immunization coverage data derived from household health surveys is higher quality than coverage data from administrative sources [60].

Data gaps:

GPEI Polio Campaign Monitoring Reports are useful for monitoring the quality of SIAs. The Decade of Vaccine Collaboration could consider expanding campaign monitoring to additional countries.

| Source | Description | Geographic scope | Years available |
| --- | --- | --- | --- |
| Levin, Ann, Sushil Howlader, Sujata Ram, Syed Mizan Siddiqui, Izaz Razul and Subrata Routh. 1999. *Case Study on the Costs*  *and Financing of Immunization Services in Bangladesh.* Special Initiatives Report No. 21. Bethesda, MD: Partnerships for Health  Reform Project, Abt Associates Inc. [44] | Authors collected immunization cost and financing data via government publications and interviews with staff of the Ministry of Health and Family Welfare, private sector, NGOs, and donors. They gathered data on internal and external financing of the EPI program from EPI headquarters, donor databases, and projects that collaborated with NGOs. Data on local government contributions to the national immunization program were obtained via a municipality survey. Furthermore, researchers carried out a survey of private health facilities in the capital to understand the role of the private sector in provision of vaccination services. Authors collected data on immunization coverage from National Coverage Evaluation Surveys. | Bangladesh | 1997-1998 |
| GPEI Polio Campaign Monitoring Reports [62] | Reports on campaigns’ target number of children under five to vaccinate and number of children missed by region during independent monitoring checks. | Select African countries and Tajikistan and Uzbekistan | Varies by country |
| WHO Measles SIAs database [63] | Contains data on results of measles supplementary immunization activities including data on activity type, age group, extent (sub-national or national), year, number of people targeted, and number and percent of people reached. | Global | 2000-2012 |
| Van Hoang et al. Cost of providing the expanded programme on immunization: findings from a facility-based study in Viet Nam, 2005*. Bulletin of the World Health Organization*. Volume 86, Number 6, June 2008, 429-434. [64] | Collected immunization financing data from health facilities in the Bavi district. Immunization costing data were also collected from the national level to estimate costs of vaccination activities in these facilities. Information on doses supplied, doses administered, and vaccine wastage rates are also presented in the paper tabulated by antigen. Vaccination coverage was estimated based on data gathered at health facilities in the Bavi district. | Viet Nam | 2005 |

# Annex References

1. Policy Cures: **G-FINDER Database.** [https://g-finder.policycures.org/gfinder_report/search.jsp]
2. PhRMA: **Pharmaceutical Industry Profiles.** Washington, D.C.: The Pharmaceutical Research and Manufacturers of America (PhRMA). [<http://www.phrma.org/profiles-reports>]
3. The Foundation Center: **IRS 990 Finder.** [http://foundationcenter.org/findfunders/990finder/]
4. Organization for Economic Co-operation and Development: **OECD- Creditor Reporting System.** [http://stats.oecd.org/Index.aspx?datasetcode=CRS1]
5. AidData: **AidData.org.** [http://aiddata.org/content/index/data-search]
6. International Aid Transparency Initiative: **IATI Registry.** [http://iatiregistry.org/dataset]
7. Bill & Melinda Gates Foundation: **Grants Database.** [http://www.gatesfoundation.org/grants/Pages/search.aspx]
8. Bill & Melinda Gates Foundation: **Annual Tax Returns.** [http://www.gatesfoundation.org/about/Pages/financials.aspx]
9. Hsu J, Pitt C, Greco G, Berman P, Mills A: **Countdown to 2015: changes in official development assistance to maternal, newborn, and child health in 2009-10, and assessment of progress since 2003.**  *Lancet* 2012, 380, no. 9848: 1157–68.
10. Institute for Health Metrics and Evaluation.  **Development Assistance for Health Estimates 1990-2010 Tables.**  Seattle, United States: Institute for Health Metrics and Evaluation, 2010. [http://ghdx.healthmetricsandevaluation.org/record/development-assistance-health-estimates-1990-2010-tables]
11. OECD Development Assistance Committee: **Reporting Directives for the Creditor Reporting System.** 2007. [http://www.oecd.org/dac/aidstatistics/1948102.pdf]
12. International Aid Transparency Initiative: **IATI Standard.** [http://iatistandard.org/]
13. Global Health Strategies Initiatives (GHSi): **Shifting Paradigm: How the BRICS Are Reshaping Global Health and Development.** New York: Global Health Strategies Initiatives, 2012. [http://www.ghsinitiatives.org/downloads/ghsi_brics_report.pdf]
14. Clinton, H R. **Keynote at the Opening Session of the Fouth High-Level Forum on Aid Effectiveness.** Busan, South Korea, 2011. [http://www.state.gov/secretary/rm/2011/11/177892.htm]
15. GAVI Alliance: **Advanced Market Commitment Documents.** [http://www.gavialliance.org/library/gavi-documents/amc/]
16. The World Bank: **Projects & Operations.** [http://www.worldbank.org/projects]
17. The World Bank. **Trust Funds Database.** [https://finances.worldbank.org/page/trust-funds]
18. Asian Development Bank: **Projects Database.** [http://www.adb.org/projects/]
19. African Development Bank Group: **Project Portfolio.** [http://www.afdb.org/en/projects-and-operations/project-portfolio/]
20. Inter-American Development Bank: **Projects Database.** [http://www.iadb.org/en/projects/projects,1229.html]
21. GAVI Alliance: **Disbursements by Country.** [http://www.gavialliance.org/results/disbursements/]
22. UNICEF Supply Division: **Vaccine Procurement 1996-2011.** [http://www.unicef.org/supply/index_vaccines.html]
23. UNICEF Supply Division: **Supply Annual Report 2011.** Copenhagen, Denmark: UNICEF, 2012. [http://www.unicef.org/supply/index_report.html]
24. Global Polio Eradication Initiative: **Financial Resource Requirements 2012-2013.** Washington, D.C.: World Health Organization, 2012. [http://www.polioeradication.org/Financing.aspx]
25. Pan American Health Organization: **Financial Report of the Director and Report of the External Auditor, 1 January 2008- 31 December 2009**. Washington, D.C.: Pan American Health Organization. [http://www2.paho.org/hq/dmdocuments/2010/FinancialReport-2008-2009.pdf]
26. World Health Organization: **Financial Report and Audited Financial Statements for the Period 1 January 2010- 31 December 2011.** World Health Organization, 2012. [http://apps.who.int/gb/ebwha/pdf_files/WHA65/A65_29-en.pdf]
27. United States Agency for International Development: **2012 VolAg Report: Report of Voluntary Agencies Engaged in Overseas Relief and Development.** Washington, D.C.: USAID, 2012. [http://dec.usaid.gov/index.cfm]
28. United States Agency for International Development (USAID) and Institute for Health Metrics and Evaluation (IHME): **IHME Formatted USAID VolAg Database 1990-2008.** Seattle, United States: Institute for Health Metrics and Evaluation (IHME), 2011.
29. Waters HR, Dougherty L, Tegang SP, Tran N, Wiysonge CS, Long K, Wolfe ND, and Burke DS. **Coverage and Costs of Childhood Immunizations in Cameroon*.*** *Bulletin of the World Health Organization* 2004, 82, no. 9: 668–75.
30. Capobianco E, Naidu V. **A Review of Health Sector Aid Financing to Somalia.** World Bank Working Paper. Africa Human Development Series. Washington, D.C.: The World Bank, 2008.
31. The World Bank: Health, Population & Nutrition Division: **Immunization Financing in Pakistan.** Washington, D.C.: The World Bank, 2000.
32. World Health Organization: **cMYP Immunization Financing Database.** World Health Organization, Immunization Financing Databases. [http://www.who.int/immunization_financing/data/en/]
33. World Health Organization: **Revisions on the cMYP Costing and Financing Tool.** Geneva, Switzerland: World Health Organization, 2012. [http://www.who.int/immunization_financing/tools/Tool_Revisions.pdf]
34. World Health Organization, Department of Immunization, Vaccines and Biologicals. **Immunization Costing and Financing: A Tool and User Guide for Comprehensive Multi-Year Planning (cMYP).** Geneva, Switzerland: World Health Organization, 2006. [http://www.who.int/immunization_financing/tools/cMYP_Costing_Tool_Manual.pdf]
35. World Health Organization: **WHO/UNICEF Joint Reporting Process.** [http://www.who.int/immunization_monitoring/routine/joint_reporting/en/index.html]
36. GAVI Alliance: **GAVI Progress Reports.** [http://www.gavialliance.org/country/]
37. UN Statistics Division: **United Nations Commodity Trade Statistics Database.** UN COMTRADE. [http://comtrade.un.org/db/mr/daCommodities.aspx]
38. International Trade Centre (ITC), Market Analysis and Research: **ITC Trade Map.** [http://legacy.intracen.org/marketanalysis/TradeMap.aspx]
39. Kaddar M, Makinen M, Khan M: **Financing Assessment Tool for Immunization Services: Guidelines for Performing a Country Assessment.** Health Reform Tools Series. Bethesda, MD: Partnerships for Health Reform Project, Abt Associates Inc, 2000.
40. Biellik R, Levin C, Mugisha E, LaMontagne DS, Bingham A, Kaipilyawar S, Gandhi S: **Health Systems and Immunization Financing for Human Papillomavirus Vaccine Introduction in Low-resource Settings.** *Vaccine* 2009, 27, no.44: 6203–9.
41. ARIVA: **ARIVA - Projet d’Appui au Renforcement de l’Indépendance Vaccinale en Afrique.** [http://www.ariva.bf/html/presentation.htm]
42. Kaddar M, Tanzi VL, Dougherty L: **Case Study on the Costs and Financing of Immunization Services in Côte d’Ivoire.** Special Initiatives Report. Bethesda, MD: Partnerships for Health Reform Project, Abt Associates Inc., 2000.
43. Kaddar M, Mookherji S, DeRoeck D, Antona D: **Case Study on the Costs and Financing of Immunization Services in Morocco.** Special Initiatives Report. Bethesda, MD: Partnerships for Health Reform Project, Abt Associates Inc., 1999.
44. Levin A, Howlader S, Ram S, Siddiqui SM, Razul I, Routh S: **Case Study on the Costs and Financing of Immunization Services in Bangladesh.** Special Initiatives Report. Bethesda, MD: Partnerships for Health Reform Project, Abt Associates Inc., 1999.
45. Asian Development Bank: Agriculture and Social Sectors Department: **Sri Lanka National Immunization Program Financing Assessment.** Asian Vaccination Initiative. Manila, Philippines: Asian Development Bank, 2001.
46. Chee G: **Cambodia Immunization Assessment: Report of Financial Findings.** Bethesda, MD: Abt Associates Inc., 2000.
47. Levin A, England S, Jorissen J, Garshong B, Teprey J: **Case Study on the Costs and Financing of Immunization Services in Ghana.** Bethesda, MD: The Partners for Health Reformplus Project, Abt Associates Inc., 2001.
48. The World Bank: Health, Population & Nutrition Division: **Immunization Financing in Pakistan.** Washington, D.C.: The World Bank, 2000.
49. *Sabin Vaccine Institute’s Sustainable Immunization Financing Initiative.* [http://www.sabin.org/updates/pressreleases/sabin-vaccine-institutes-sustainable-immunization-financing-initiative]
50. World Health Organization: **JRF Immunization Financing Indicators Database.** [http://www.who.int/immunization_financing/data/en/index.html]
51. Fort A : **Service Provision Assessment.** New York, United States: UNFPA. [http://www.unfpa.org/webdav/site/global/shared/documents/publications/2010/srh_guide/tools_serviceprovision.html]
52. Measure DHS: **Demographic and Health Surveys.** [http://www.measuredhs.com/What-We-Do/survey-search.cfm?pgType=main&SrvyTp=type]
53. Levin A, Kaddar M: **Role of the Private Sector in the Provision of Immunization Services in Low- and Middle-income Countries.** *Health Policy and Planning* 2011, 26: i4– i12.
54. Ebong C, Levy P: **Impact of the Introduction of New Vaccines and Vaccine Wastage Rate on the Cost-effectiveness of Routine EPI: Lessons from a Descriptive Study in a Cameroonian Health District.** *Cost Effectiveness and Resource Allocation* 2011, 9, no. 9. doi:10.1186/1478-7547-9-9.
55. Lydon P: **Costing of Measles Campaigns West Pacific Region: The Lao PDR Measles Campaigns 2000-2001.** Geneva, Switzerland: World Health Organization.
56. Measure DHS: **Demographic and Health Surveys (DHS).** [http://www.measuredhs.com/Data/]
57. UNICEF, Childinfo: **Multiple Indicator Cluster Surveys (MICS).** [http://www.childinfo.org/mics.html]
58. World Health Organization and UNICEF: **Reported Estimates of Immunization Coverage Time Series.** [http://www.who.int/immunization_monitoring/data/data_subject/en/index.html]
59. World Health Organization and UNICEF: **Estimates of Immunization Coverage Time Series.** [http://www.who.int/immunization_monitoring/data/data_subject/en/index.html]
60. Lim SS, Stein DB, Charrow A, Murray CJ: **Tracking Progress Towards Universal Childhood Immunisation and the Impact of Global Initiatives: a Systematic Analysis of Three-dose Diphtheria, Tetanus, and Pertussis Immunisation Coverage.** *Lancet* 20087, 372, no. 9655: 2031–2046.
61. World Health Organization: **Data, Statistics and Graphics by Subject.** [http://www.who.int/immunization_monitoring/data/data_subject/en/index.html]
62. Global Polio Eradication Initiative: **Global Polio Campaign Monitoring Reports.** Geneva, Switzerland: Global Polio Eradication Initiative, 2011. H[ttp://www.polioeradication.org/Dataandmonitoring/Poliocampaignmonitoring.aspx]
63. World Health Organization: **Retrospective Measles Data on Supplementary Immunization Activities, 2000-2011.** Supplementary Immunization Activities, World Health Organization. [http://www.who.int/immunization_monitoring/data/data_subject/en/index.html]
64. Van Hoang M, Yen Nguyen TB, Giang Kim B, Huong Dao L, Huong Nguyen T, Wright P. **Cost of Providing the Expanded Programme on Immunization: Findings from a Facility-based Study in Viet Nam, 2005.** *Bulletin of the World Health Organization* 2008, 86, no. 6: 429–434.
